# Results of off‐pump coronary artery bypass grafting with off‐pump first strategy in octogenarian

**DOI:** 10.1111/jocs.16055

**Published:** 2021-10-06

**Authors:** Hideki Kitamura, Mototsugu Tamaki, Yasuhiko Kawaguchi, Yasuhide Okawa

**Affiliations:** ^1^ Department of Cardiovascular Surgery Nagoya Heart Center Nagoya Aichi Japan

**Keywords:** coronary artery disease, off‐pump coronary artery bypass grafting, octogenarian

## Abstract

**Background and Aim:**

Ischemic heart disease is the leading cause of death around the world. Coronary artery bypass grafting offers efficient surgical revascularization for ischemic disease. Both on‐ or off‐pump coronary artery bypass methods provide promising results to octogenarians, once complete vascularization is achieved. However, off‐pump bypass requires a certain level of experience to achieve sufficient results. We have applied an off‐pump coronary artery bypass‐first strategy to all generations since 2008. This study investigated early and long‐term results of surgical revascularization for octogenarians by a team with an off‐pump‐first strategy.

**Methods:**

All cases of isolated coronary artery bypass grafting performed since 2008 were identified and divided into a young group (age < 80 years) and an old group (age ≥ 80 years). Peri‐operative results were investigated retrospectively in both groups and long‐term results for the old group were assessed.

**Results:**

Among the 707 patients, 97% underwent off‐pump bypass, and 94 cases were classified to the old group. Distal anastomoses and ventilator time were identical between groups (young vs. old: 3.3 vs. 3.2; 3.7 h vs. 3.7 h). In‐hospital death rates were 0.5% and 0% in the young and old groups, respectively. With a mean follow‐up of 1318 days, actual 1‐, 3‐, and 5‐year survival rates for octogenarians were 92.1%, 81.2%, and 68.3%, respectively. Nearly half of the patients reached their nineties, which was close to the life expectancy of the national general octogenarian.

**Conclusions:**

An experienced team with an off‐pump‐first strategy could provide valid therapeutic options for octogenarians.

## 1 INTRODUCTION

Ischemic heart disease is the leading cause of death around the world.^1^ As the global population increases, so is the proportion of elderly individuals.^2^ This is particularly prominent in Japan, where the average life expectancy is over 80 years for both sexes, and the proportion of elderly is high.^3^ As a result, more and more elderly individuals with ischemic heart disease are being encountered.

Older patients reportedly show more benefits from any type of revascularization than from medical therapy, in terms of not only symptom relief and quality of life, but also long‐term survival.^4^, ^5^

Compared with percutaneous coronary intervention, coronary artery bypass grafting (CABG) shows higher mortality and morbidity rates as early results.^6^ However, the long‐term results are better than those from percutaneous coronary intervention.^6^ Considering that life expectancy at 80 years old is around 10 years for the Japanese general population,^3^ long‐term results should be taken into consideration even among octogenarians. CABG may thus be applicable to elderly patients where feasible, but improvements in early results are a priority.

Off‐pump CABG (OPCAB) eliminates the use of cardiopulmonary bypass, and so might benefit high‐risk patients or complex cases with concomitant disease.^7^ However, whether OPCAB is better for octogenarian than conventional CABG remains controversial.^7^, ^8^, ^9^, ^10^ Both methods provide promising early and midterm results to octogenarians, once complete vascularization is achieved.^10^ However, OPCAB seems to require a certain amount of experience to achieve sufficient results compared to conventional under‐arrest CABG.^11^ In our institution, OPCAB is the first choice for surgical revascularization of isolated CABG cases in all generations. Both surgeons and co‐workers are very familiar with the management of OPCAB.

The purpose of this study was to elucidate peri‐operative results of OPCAB in octogenarians with our OPCAB‐first strategy and to investigate differences between octogenarian and younger patients. We also explored the long‐term results for elderly patients and evaluated the validity of the OPCAB‐first strategy among octogenarians.

## 2 MATERIALS AND METHODS

This study complied with the Declaration of Helsinki and was approved by Nagoya Heart Center Ethics Committee (approved number: NHC2021‐0330‐12) on March 30, 2021. The need to obtain written informed consent was waived because of retrospective design of the study by Nagoya Heart Center Ethics Committee. Between October 2008 and February 2021, all cardiac surgery cases performed at Nagoya Heart Center were screened and cases of isolated CABG were identified. We then divided patients into two groups: less than 80 years old (young group); and more than or equal to 80 years old (old group). Peri‐operative results were investigated and compared between groups. Clinical data were gathered from medical charts, operative records and the in‐hospital surgical database. Long‐term results were assessed by direct contact or telephone interviews with patients, their families, or local doctors. Survival was assessed from January to May 2021.

The primary endpoint of this study was to investigate peri‐operative results of the old group and compare them with those of the young group. The secondary endpoint was to assess long‐term survival in the old group.

Data were analyzed using SPSS version 22 statistical software (SPSS). Results are expressed as mean ± standard deviation. Continuous data were analyzed using the Mann–Whitney U test or *t*‐test, as appropriate. Categorical data were analyzed using the *χ* ^2^ test. Survival analysis was performed using the Kaplan–Meier method. Results were considered significant for values of *p* < .05.

### 2.1 Patient selection, operative strategy, and procedures

We have a no‐refusal policy for surgical revascularization, although some exclusion criteria are applied. One is for patients with mental disorders such as schizophrenia or severe dementia. Another is for very fragile or bedridden patients. We regard three factors as important in achieving recovery from the surgery: the ability to take a deep breath and cough; the ability to eat well; and the ability to stand and walk. We, therefore, do not recommend surgical therapy for bedridden or severely fragile patients. We do not apply any upper limit on age for the surgery, but if the patient cannot perform the abovementioned three activities, we do not recommend the surgery, even for patients younger than 80 years old.

OPCAB is the first‐choice procedure for isolated CABG in our institution. Cardiopulmonary bypass was used when maintaining adequate hemodynamics proved difficult or when hemodynamic instability was anticipated. Our standard strategy for surgical coronary artery revascularization is to use bilateral internal thoracic arteries (ITAs) where possible and to perform bypass with these vessels to the left coronary artery system.^12^ However, in cases of moderate stenosis of the circumflex artery, saphenous vein graft is considered to avoid graft competition.^13^ In cases of very ill patients, we avoid bilateral ITA usage and choose saphenous vein grafts to shorten the operative time. The ITA is harvested in skeletonized fashion with a harmonic scalpel by experienced surgeons.

The great saphenous vein is mainly used as a graft to the right coronary artery. However, when the ascending aorta has a severe atherosclerotic disease, the gastroepiploic artery is used to avoid manipulation of the aorta.^14^ To anastomose the proximal site of the saphenous vein graft to the ascending aorta, clampless facilitating devices (either Heart String or Enclose, depending on surgeon preference) are routinely used.

### 2.2 Preoperative care

As preoperative assessment, swallowing, and coughing were judged as well as physical activity assessment for the patient. Given the high risk of aspiration pneumonia, a physical therapist taught each patient how to cough efficiently and a nutritionist arranged the diet (such as adding viscosity to prevent aspiration).

Evaluation of cardiac function was performed by transthoracic echocardiogram; cardiac chamber dimensions were measured by the standard M‐mode method and ejection fraction was calculated by the modified Simpson's method. Mitral valve regurgitation was assessed using color‐flow Doppler as “trivial,” “mild,” “moderate,” or “severe” which correspond “1+”, “2+”, “3+,” or “4+”, respectively.

### 2.3 Intraoperative care

During off‐pump surgeries, stable hemodynamics are crucial. We opened the pericardium in an inverted‐T fashion and added a vertical incision to the inferior surface to expand the pericardial cavity in the lateral dimension. This prevents right‐side heart kinking when located in a standing position. We used phenylephrine as needed to maintain systemic blood pressure and excessive volume infusion was avoided where possible, to prevent postoperative edema or deterioration of oxygenation. Hemodynamics are monitored with Swan‐Ganz catheter including pulmonary artery pressure, mixed venous oxygen saturation and cardiac output.

General anesthesia was maintained by inhaled anesthesia after anesthetic induction. As a muscle relaxant, rocuronium bromide was continuously infused at a specific dose according to the bodyweight of the patient, and was reversed with sugammadex when postoperative hemodynamic stability was confirmed.

### 2.4 Postoperative care

Our standard postoperative protocol was as follows. The patient was transferred from the intensive care unit to the general ward on postoperative day (POD) 1. On the same day, oral intake was initiated, caloric intake was assessed, and further diet was arranged by the nutritionist.

We started oral aspirin at 81 mg/day on POD1, and clopidogrel at 75 mg/day following drainage tube removal (usually POD2).^15^

Concerning rehabilitation, the patient attempted to reach a standing position and to take steps with the physical therapist on POD1. This was advanced to a 100‐m walk in 10 min on an ergometer on POD2, and a 300‐m walk in 10 min on the ergometer on POD3.

Grafts were evaluated before discharge by coronary angiography or contrast‐enhanced computed tomography.

## 3 RESULTS

During the study period, 2978 cases of cardiac surgery were performed in our institution, of which 707 cases were identified as isolated CABG (687 cases [97.2%] were off‐pump). Of these, 94 cases were more than 80 years old at the time of surgery. Table 1 shows the preoperative characteristics of the 613 cases in the young group and 94 cases in the old group. The old group showed a higher proportion of females, smaller body size and lower frequencies of diabetes, dyslipidemia, and smoking history compared with the young group. The older group displayed lower renal function and higher frequency of anemia compared to the young group. Euroscore II and Clinical Frailty Scale were significantly higher in the old group than in the young group. The old group displayed a higher frequency of left main trunk disease than the young group, but preoperative echocardiographic findings were broadly comparable. Table 2 shows the operative results. The old group had a shorter operative time, lower frequency in the usage of right internal thoracic arteries (ITAs), and a greater frequency of transfusion than the young group. The number of distal anastomoses was identical between groups. Cardiopulmonary bypass was used for 3.3% of cases in the young group, whereas all cases in the old group were treated using OPCAB, as intended. Graft patency rate was 97.0% in the young group and 96.2% in the old group. Postoperative hospitalization was longer in the old group, but ventilator time and stay in the intensive care unit were comparable between groups. Eighty of 94 old patients (85.1%) were discharged to home. Fourteen patients were transferred to another hospital, with 13 for further rehabilitation, and one for treatment of cerebral infarction. Operative/in‐hospital death rates were very low in both groups (young group, 0.5%; old group, 0%).

**Table 1:** Preoperative characteristics in young and old groups

Variable	Young (*n* = 613)	Old (*n* = 94)	*p* value
Mean age (years)	66.4 ± 9.8	82.9 ± 2.7	<**.001**
Male (*n*)	509 (83.0%)	61 (64.9%)	<**.001**
Body surface area (m^2^)	1.70 ± 0.18	1.55 ± 0.14	<**.001**
Body mass index (kg/m^2^)	24.1 ± 3.5	22.5 ± 2.5	<**.001**
EuroScore II	2.9 ± 4.4	5.9 ± 6.0	<**.001**
Clinical Frailty Scale	2.8 ± 0.7	3.2 ± 0.9	<**.001**
Emergent (*n*)	71 (11.6%)	14 (14.9%)	.358
Preoperative IABP (*n*)	85 (13.9%)	13 (13.9%)	.988
Preoperative intubation (*n*)	2 (0.4%)	0 (0%)	.765
Hypertension (*n*)	404 (65.9%)	64 (68.1%)	.677
Dyslipidaemia (*n*)	455 (74.2%)	60 (63.8%)	.051
Diabetes (*n*)	326 (58.1%)	38 (40.4%)	**.021**
Prior cerebrovascular disease (*n*)	34 (5.5%)	2 (2.1%)	.160
Peripheral artery disease (*n*)	105 (17.1%)	24 (25.5%)	**.050**
Prior cardiac surgery	11 (2.2%)	3 (3.2%)	.365
Prior percutaneous coronary intervention	149 (24.3%)	18 (26.6%)	.631
Old myocardial infarction	134 (21.9%)	20 (21.3%)	.899
COPD (*n*)	111 (18.1%)	24 (25.5%)	.088
Smoking history (*n*)	410 (66.9%)	50 (53.2%)	**.010**
Hemodialysis (*n*)	89 (14.5%)	8 (8.5%)	.115
eGFR (ml/min/1.73 m^2^)	54.5 ± 25.2	50.2 ± 20.5	**.015**
Hematocrit (%)	39.5 ± 5.0	36.0 ± 5.8	<**.001**
Echocardiographic data			
Ejection fraction (%)	54.8 ± 14.7	53.5 ± 13.8	.941
Left atrial diameter (mm)	38.0 ± 5.8	38.0 ± 5.4	.936
Left ventricular diastole diameter (mm)	48.1 ± 6.7	45.7 ± 5.6	**.002**
Left ventricular systole diameter (mm)	34.2 ± 8.6	32.8 ± 7.5	.399
Mitral regurgitation	1.3 ± 0.8	1.5 ± .07	.053
Coronary angiographic data			
Left main trunk disease (*n*)	194 (31.6%)	42 (44.7%)	**.013**
Three‐vessel disease (*n*)	475 (77.5%)	73 (77.7%)	.970

Abbreviations: COPD, chronic obstructive pulmonary disease; eGFR, estimated glomerular filtration rate; IABP, intra‐aortic balloon pump.

**Table 2:** Operative results in young and old groups

Variable	Young (*n* = 613)	Old (*n* = 94)	*p* value
Operation time (min)	252 ± 69	235 ± 64	**.016**
Cardiopulmonary bypass (*n*)	20 (3.3%)	0 (0%)	.076
Distal anastomosis	3.3 ± 1.1	3.2 ± 1.0	.100
Left internal thoracic artery (*n*)	598 (97.6%)	90 (95.7%)	.313
Right internal thoracic artery (*n*)	510 (83.2%)	70 (74.5%)	**.040**
Bilateral internal thoracic arteries (*n*)	497 (81.1%)	67 (71.3%)	**.028**
Saphenous vein graft (*n*)	463 (75.5%)	78 (83.0%)	.113
Gastroepiploic artery (*n*)	26 (4.2%)	1 (1.1%)	.134
Transfusion (*n*)	275 (44.9%)	74 (78.7%)	<**.001**
Graft evaluation (*n*)	574 (93.6%)	86 (91.5%)	
Graft patency	97.0%	96.2%	
Ventilator time (h)	3.7 ± 3.9	3.7 ± 3.3	.610
Intensive care unit stay (days)	1.2 ± 1.3	1.5 ± 3.2	.248
Post‐op. hospitalization (days)	12.6 ± 8.7	14.8 ± 7.3	<**.001**
Readmission within 30 days (*n*)	11 (1.8%)	3 (3.2%)	.370
Operative/in‐hospital death (*n*)	3 (0.5%)	0 (0%)	.496
Re‐intubation (*n*)	17 (2.8%)	2 (2.1%)	.719
Re‐exploration (*n*)	8 (1.3%)	2 (2.1%)	.846
Mediastinitis (*n*)	5 (0.8%)	1 (1.1%)	.807
Cerebral infarction (*n*)	11 (1.8%)	2 (2.1%)	.823
Require temporary hemodialysis (*n*)	6 (1.0%)	6 (6.3%)	<**.001**
Post‐op. atrial fibrillation (*n*)	125 (20.4%)	34 (36.2%)	**.001**

Abbreviation: Post‐op, postoperative.

The old group required temporary postoperative hemodialysis and suffered atrial fibrillation more frequently than the young group, but no significant differences were seen in other morbidities, including re‐intubation, re‐exploration, mediastinitis, or cerebral infarction.

**Figure 1:**
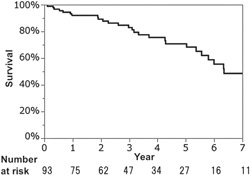
Kaplan–Meier analysis of survival from all‐cause death

The collection rate for follow‐up data was 100%, with a mean follow‐up of 1318 days (range, 40–4309 days). Figure 1 shows the survival curve for all‐cause death. Actual 1‐, 3‐, and 5‐year survival rates were 92.1%, 81.2%, and 68.3%, respectively. Twenty‐eight deaths occurred during the observational period. Leading causes of late death were infection, including pneumonia (*n* = 7). Other causes of death were heart failure in five cases, neurological and natural death (death from old age) in four cases each, cancer in three cases, and renal failure in two cases.

## 4 DISCUSSION

This study was a retrospective, single‐center report of early and long‐term results from OPCAB for octogenarians under an OPCAB‐first strategy and illustrated real‐world clinical practice. Early results were good in terms of mortality and morbidity rates, with the exception of postoperative atrial fibrillation. Long‐term results were also good.

As postoperative results for the old group were not inferior to those for the young group, we believe that the OPCAB‐first strategy for all generation becomes a good therapeutic option for surgical revascularization in octogenarians.

For elderly patients, early extubation and early recovery from bed rest are very important to prevent disuse atrophy, which can lead to postoperative complications. In the present study, postoperative intubation time and intensive care unit stay did not differ significantly between young and old groups. We adjusted graft usage in elderly or ill patients to shorten operative time, and the old group displayed a shorter operative time than the young group (Table 2). Shorter operative time and OPCAB with multidisciplinary care, including anesthetic drug usage and intraoperative volume control could contribute to the achievement of early extubation and a short stay in the intensive care unit, even for old patients.

Some reports have demonstrated that bilateral ITA usage was associated with an increased frequency of postoperative mediastinitis.^16^, ^17^ In the present study, however, the overall incidence of mediastinitis was low, at 0.8%, despite bilateral ITAs were used in 79.8% of all cases. This might be because bilateral ITAs were harvested in skeletonized fashion with a harmonic scalpel in our institution.^18^ Furthermore, the surgeons who harvested bilateral ITAs were very experienced due to our strategy, and bilateral ITAs were used in nearly 80% of all cases.

Concerning the graft design, the radial artery was attractive as the third arterial graft.^14^ However, in the present study, nearly 14% of patients were receiving hemodialysis before the operation, and radial artery harvesting was thus avoided in consideration of the fact that many patients with coronary artery disease potentially have reduced renal function and even nonhemodialysis patients might require future shunt establishment. As a matter of fact, 6.3% of the old group required temporary postoperative hemodialysis, even though OPCAB can reportedly lower postoperative kidney injury compared to conventional CABG.^19^, ^20^

The frequency of postoperative cerebral infarction did not differ between groups, but was 1.8% overall. Three of the 13 patients with postoperative cerebral infarction were considered to have intraoperative cerebral infarction, comprising two emergent cases, and one cases with carotid stenosis. The “intra‐operative” cerebral infarction rate was low, at 0.4%, probably because OPCAB could avoid aortic cannulation or cross‐clamping.^11^ The remaining 10 cases suffered delayed cerebral infarction on POD3 or later, including five cases of paroxysmal atrial fibrillation, and three cases in hemodialysis patients. Methods to reduce “postoperative” cerebral infarction are needed. We adopted double antiplatelet therapy, with aspirin at 81 mg/day and clopidogrel at 75 mg/day, with the aims of not only reducing graft occlusion,^15^ but also reducing the risk of cerebral infarction.^21^, ^22^ However, our results indicate that changes in this postoperative regimen may be warranted. Adjustment of antiplatelet/anticoagulation therapy, including heparin administration or use of warfarin or direct oral anticoagulants, might be required. Not only care, but also prevention of atrial fibrillation are the rational approaches to reducing postoperative cerebral infarction. This needs to be taken into consideration, using approaches such as aggressive usage of beta‐blockers.^23^

Long‐term results were satisfactory considering the age of our patients, and were better than what has been reported elsewhere.^4^, ^5^ These findings were comparable with those from previous reports; once old patients survive the surgery, long‐term prospects are good.^6^ In the present study, the 7‐year survival rate was 48.6%, meaning that nearly half of the patients reached their nineties. This was close to the life expectancy of the general population in Japanese at age 80, which is about 9 years for Japanese men and 12 years for Japanese women.^3^

The Clinical Frailty Scale is a simple tool to semi‐quantitatively assess patient frailty.^24^ This scale can predict late mortality in certain cases.^25^, ^26^ In the present study, as preoperative Clinical Frailty Scale scores differed significantly between groups, the mean score in the old group was relatively low, at 3.2. Despite our no‐refusal policy, some degree of selection bias would have naturally been introduced by referring doctors, cardiologists or even surgeons. This speculation was supported by the fact that 85.1% of patients were able to return home from hospital directly, indicating that the daily activity of octogenarian patients was well preserved in this study. From another perspective, our results implied that those patients with a Clinical Frailty Scale score around 3 would have a high possibility of overcoming OPCAB, even in octogenarians.

The present study had several limitations. First, the results in this study represent our clinical experience with a consecutive series of isolated surgical CABG. The number of patients was small and selection of surgical revascularization for patients introduced a huge selection bias. Nonetheless, our results encourage us to keep providing open‐heart surgery to octogenarians with ischemic coronary disease where feasible.

A second limitation was the retrospective design of the study, with no comparisons between OPCAB and conventional CABG made within groups. Whether OPCAB provided more benefit to old patients than conventional CABG thus could not be assessed. However, as we chose OPCAB as the first‐line procedure, nearly 97% of cases were accomplished using OPCAB. We believe that the more we become familiar with the procedure, the more the operative results that can be provided to elderly patients will stabilize.^11^

Lastly, we did not assess major adverse cardiac events over the long term. The long‐term symptom‐free rate was therefore unknown. Nevertheless, our OCPAB‐first strategy could provide elderly patients with a comparable survival rate to Japanese octogenarians in the general population.

## 5 CONCLUSIONS

In conclusion, both early and long‐term results of OPCAB for octogenarians were good. The OPCAB‐first strategy could represent a valid therapeutic option for octogenarians. However, Clinical Frailty Scale score for octogenarians in the study was low, so surgical indications should be considered carefully. Further accumulation of data and experiences is mandatory.

## CONFLICT OF INTERESTS

The authors declare that there are no conflict of interests.

## AUTHOR CONTRIBUTIONS

Hideki Kitamura contributed the article design, concept, drafting and revision of the article. Yasuhide Okawa contributed the article design, concept, and data interpretation. Mototsugu Tamaki and Yasuhiko Kawaguchi contributed data collection and data analysis.
